# Smart Pasta Design: Tailoring Formulations for Technological Excellence with Sprouted Quinoa and Kiwicha Grains

**DOI:** 10.3390/foods13020353

**Published:** 2024-01-22

**Authors:** Luz María Paucar-Menacho, Marcio Schmiele, Juan Carlos Vásquez Guzmán, Sander Moreira Rodrigues, Wilson Daniel Simpalo-Lopez, Williams Esteward Castillo-Martínez, Cristina Martínez-Villaluenga

**Affiliations:** 1 Departamento Académico de Agroindustria y Agronomía, Facultad de Ingeniería, Universidad Nacional del Santa, Chimbote 02712, Peru; luzpaucar@uns.edu.pe (L.M.P.-M.); jvasquez@uns.edu.pe (J.C.V.G.); wsimpalol@uns.edu.pe (W.D.S.-L.); wcastillo@uns.edu.pe (W.E.C.-M.); 2 Institute of Science and Technology, Federal University of Jequitinhonha and Mucuri Valleys, Diamantina 39100-000, Brazil; marcio.sc@ict.ufvjm.edu.br (M.S.); sander.moreira@ufvjm.edu.br (S.M.R.); 3 Department of Technological Processes and Biotechnology, Institute of Food Science, Technology and Nutrition (ICTAN), Spanish National Research Council (CSIC), 28040 Madrid, Spain

**Keywords:** sprouted pseudocereals, formulation, cooking, pasta, texture, color, optimization

## Abstract

The pursuit of developing healthier pasta products without compromising technological properties involves a strategic approach via the customization of raw material formulations and the integration of grain germination and extrusion processes. This study explores the impact of incorporating sprouts from quinoa (*Chenopodium quinoa* Willd) and kiwicha (*Chenopodium pallidicaule* Aellen) on the physicochemical properties of pasta by employing a centroid mixture design. The desirability function was utilized to identify the optimal ingredient proportions necessary to achieve specific objectives. The study identified optimal formulations for two pasta variations: pasta with the substitution of sprouted quinoa and cushuro powder (PQC), and pasta with partial substitution of sprouted kiwicha and cushuro powder (PKC). The optimal formulation for PKC was determined as 70% wheat flour (WF), 15% sprouted kiwicha flour (SKF), and 15% cushuro powder (CuP), with a desirability score of 0.68. Similarly, for PQC, the optimal formulation comprised 79% WF, 13% sprouted quinoa flour (SQF), and 8% CuP, with a desirability of 0.63. The optimized pasta formulation exhibited longer cooking times (10 and 8 min), increased weight gain (235% and 244%), and minimal loss of solids (1.4 and 1.2%) for PQC and PKC, respectively. Notably, firmness (2.8 and 2.6 N) and breaking strength values (2 and 2.7 N) for PQC and PKC pasta formulations, respectively, were comparable to those of the control sample (2.7 N and 2.6 N for firmness and fracturability, respectively). This research underscores the potential of tailored formulations and innovative processes to enhance the nutritional profile of pasta while maintaining key technological attributes.

## 1. Introduction

Pasta stands among the most widely consumed foods globally, cherished for its affordability, widespread acceptance, delightful taste, and convenient preparation and storage. Traditionally crafted from wheat flour or semolina, pasta serves as a comforting energy source with a relatively low glycemic index but a high glycemic load. It is deficient in vitamins, minerals, dietary fibers, amino acids, and phytochemical compounds that confer potential health benefits [1,2]. Recognizing the need for nutritional enhancement, recent attention has focused on utilizing unconventional raw materials to develop healthier pasta alternatives [1].

Several studies have demonstrated that partially replacing wheat flour with pseudocereal flour, including amaranth, buckwheat, and quinoa, significantly augments the nutritional value of pasta [1,3]. These pseudocereal-enriched pastas, whether composed solely of pseudocereal flours or in combination with wheat flour or semolina, boast elevated levels of micronutrients and phytonutrients, along with a more balanced amino acid profile compared to traditional cereals like rice, wheat, and corn [4]. Additionally, the use of pseudocereals favors the nutritional profile of foods, increasing the content of vitamins, minerals, and dietary fibers.

There is a burgeoning interest in exploring flours derived from sprouted grains due to their enhanced nutritional value and bioactive potential. The germination process of pseudocereal grains not only enhances the concentration and accessibility of nutrients [5,6,7], but also diminishes the presence of antinutritional factors [8,9]. This transformative process has been associated with heightened levels of bioactive compounds, including phenolic compounds, γ-aminobutyric acid, and increased antioxidant activity [10,11,12]. Beyond nutritional considerations, the flavor and texture of sprouted grains are expected to vary due to processing conditions and genetic factors. The inclusion rate of sprouted grains in formulations becomes crucial, impacting the final product’s attributes according to the food manufacturer’s preferences for flavor and texture.

Furthermore, unconventional ingredients like cushuro, a protein-rich cyanobacterium containing substantial amounts of iron and calcium, show promise in addressing issues of anemia and malnutrition [13]. Cushuro, scientifically known as *Nostoc sphaericum* Vaucher ex Bornet & Flahault, is an Andean microalga thriving in macrocolonies at altitudes exceeding 3000 m in the Andean foothills. These colonies can be manually harvested, sun-dried, and then marketed locally [14]. Although traditionally a dietary staple in specific South American areas, this microalgae has recently regained attention for its potential incorporation into restaurant menus and the creation of healthier food products in the food industry, presenting economically viable alternatives [13].

A partial or total substitution of wheat semolina with other ingredients can result in technological impacts, primarily due to the dilution of proteins responsible for the development of the three-dimensional network known as the gluten network. Dietary fibers can cause steric hindrance in the viscoelastic network, affecting the integrity and tenacity of the protein-starch network, which can lead to an increase in leached solids, impacting cooking time, weight gain, color changes, and flavor alterations, which are all critical aspects of quality control. Additionally, texture parameters must be maintained, as consumers prefer pasta to remain intact and firm after cooking, achieving the desired “al dente” texture [15,16]. The texture characteristics of pasta dough can play a crucial role in determining final consumer acceptance, indicating a preference for pastas that maintain their texture characteristics after cooking. Hence, this research seeks to achieve the optimal technological formulation for a functional pasta, featuring the partial replacement of wheat flour with sprouted Andean pseudocereal flour enriched with cushuro.

On the other hand, the proper utilization of innovative flours in pasta formulation can yield promising results. Despite the well-established understanding of the three-dimensional network formed by gluten, encompassing disulfide and hydrogen bonds, electrostatic and hydrophobic interactions, and induced dipole–dipole forces, the incorporation of novel protein sources can alleviate the deleterious effects from a technological standpoint in pasta. In addition to enhancing technological quality, benefits may extend to improvements in nutritional composition and physiological advantages for consumers’ wellbeing [17]. Thus, it is crucial to consider regulatory policies, environmental impacts, sustainability, and innovation, while also understanding consumer opinions and expectations. Therefore, assessing the technological quality of products is essential to achieve all goals, thereby ensuring the commercial success of pasta formulated with diverse raw materials [18].

There is no data in the scientific literature on the use of germinated quinoa and kiwicha with the addition of cushuro in pasta. Thus, understanding the technological behavior of these raw materials in relation to the technological effects of the pasta has stood out for its innovative nature. Hence, this research seeks to achieve the optimal technological formulation for a functional pasta, featuring the partial replacement of wheat flour with sprouted Andean pseudo-cereal flour enriched with cushuro. In this study, quinoa- and kiwicha-sprouted grains, as well as cushuro, were chosen for their nutritional richness and the potential to contribute to improved health benefits in the pasta. The choice of these raw materials was grounded in their documented positive impact on enhancing the nutritional quality of food products, particularly in terms of macronutrient digestibility, content of fiber, protein, polyunsaturated fatty acids, minerals, and bioaccessibility of bioactive compounds [19].

## 2. Materials and Methods

### 2.1. Materials

In this research, we integrated two distinct pseudocereal grains—quinoa (*Chenopodium quinoa* Willd.) and kiwicha (*Amaranthus caudatus* L.). These grains were sourced from the Cereals and Native Grains Program in Lima, Peru. Additionally, Cushuro (*Nostoc sphaericum* Vaucher ex Bornet & Flahault) was acquired from a local market in Chimbote, Peru. To enhance the functional properties of sprouted pseudocereal flours and maximize the content of bioactive compounds (γ-aminobutyric acid and phenolic compounds) along with antioxidant activity [6,7], quinoa and kiwicha grains underwent germination using an optimized procedure. Subsequently, the sprouted grains and cushuro were subjected to drying in a climatic chamber at 60 °C for 24 h. The resulting dried sprouts and cushuro, maintaining 10–12% moisture, were further processed using an MDNT-60XL grinding module and sieved through a 0.20 mm pore size sieve (Torrh, Jarcon del Peru S.R.L., Junín, Peru). This milling process yielded three raw materials for pasta production: sprouted quinoa flour (SQF), sprouted kiwicha flour (SKF), and cushuro powder (CuP). These ingredients were carefully stored at 4 °C under vacuum in plastic bags. Additionally, for comparative purposes, commercial durum wheat flour (WF, Nicolini, Alicorp S.A., Callao, Peru) was procured from the Lima market in Peru.

### 2.2. Pasta Making

In the pasta production process, our investigation focused on two types of composite flours. Composite flour 1 comprised a mixture of refined wheat flour (WF), sprouted quinoa flour (SQF), and cushuro powder (CuP) (Table 1). On the other hand, composite flour 2 was a blend of WF, sprouted kiwicha flour (SKF), and CuP (Table 2). The reference pasta (control) was crafted using 100% refined wheat flour. Subsequently, pasta samples derived from composite flour 1, composite flour 2, and refined WF were labeled as PQC, PKC, and C, respectively.

To optimize the composite flour formulations, we devised 14 recipes involving the partial substitution of WF with varying ratios of SQF and CuP (PQC, Table 1) and SKF and CuP (PKC, Table 2) using a simplex centroid mixture design.

**Figure 1:**
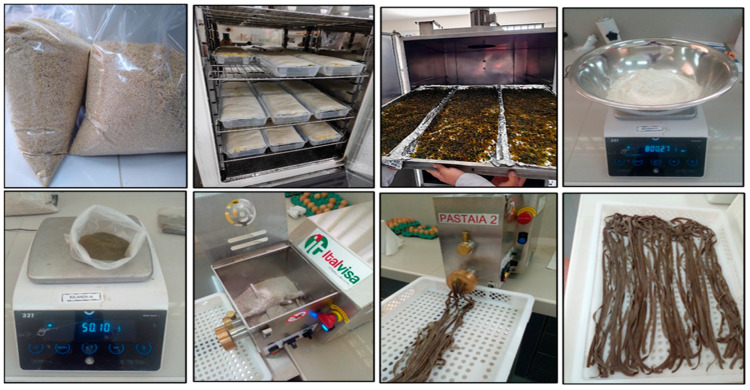
Pasta production workflow. From left to right: top: weighing and germinating grains; cushuro drying; flour weighing. At the bottom: extrusion and cutting of extruded product; finished product.

The production workflow of the pasta is illustrated in Figure 1. For dough preparation (300 g), a mixture of 180 g of composite flour (12% moisture), 60 g of hen eggs, and 60 g of water was prepared. The resulting doughs underwent kneading for 10 min at 100 rpm in a mixing bowl and were then allowed to rest in a plastic bag for 30 min. The prepared doughs (with 35% moisture) were extruded and cut into fettuccine using a Pastaia 2 extruder (Italvisa Maquinas LTDA, Tatui, Brazil). The fresh pasta was then dried on trays using natural air convection (Falc Oven Model STE-F 52, Treviglio, Italy) at 55 °C until a moisture level of 11% was achieved. Subsequently, the dried pasta was stored at 4 °C in vacuum-sealed plastic bags.

### 2.3. Simplex Centroid Mixture Design

To optimize the formulation for pasta production, a simplex centroid mixture design was employed, incorporating three ingredients, WF (A), SQF/SKF (B), and CuP (C), as independent variables. In an experiment involving q components, the proportions of the ingredients were denoted by x_1_, x_2_, …, x_q_, where x_i_ ≥ 0 for i = 1, 2, …, q and ∑q_i_ = 1x_i_ = 1. Here, x_i_ represents the proportion of the i-th component. This equation ensures that the proportions sum to 1, thereby removing a degree of freedom from the proportions. Consequently, the factor space is a (q − 1)-dimensional regular simplex [20]. The experimental conditions and coded values for the factorial design are detailed in Table 1 and Table 2.

The design allowed us to approximate the experimental data (Yobs) using a response surface model described by Equations (1)–(3):

Linear ŷ = ∑q_i_ = 1β_i_x_i_, (1)

Quadratic ŷ = ∑q_i_ = 1β_i_x_i_ + ∑_q−1_^i < j^∑q_j_β_ij_x_i_x_j_, (2)

Special cubic ŷ = ∑q_i_ = 1β_i_x_i_ + ∑_q−1_^i < j^∑q_j_β_ij_x_i_x_j_ + ∑_q−2_^i < j^∑_q−1_^j < k^∑q_k_β_ij_kx_i_x_j_x_k_, (3)

The parameter β_i_ signifies the expected response to the pure blend x_i_ = 1 and x_j_ = 0 when j ≠ i. The term ∑q_i_ = 1β_i_x_i_ accounts for the linear ingredient ratio. In cases where curvature arises due to non-linear blending between pairs of components, the parameters βij, indicating either synergistic or antagonistic blending, will deviate from zero.

The discrepancy between the experimental data (Y_obs_) and the model predictions (Y_calc_) is quantified by the residual (ε). For each response, the squared correlation coefficient (R^2^) was computed, representing the proportion of variation in the response explained by the model. In this study, a coefficient of determination greater than 0.8, a significance level less than 0.10, and a ratio F_cal_/F_tab_ ≥ 3.0 were adopted to ensure robust predictions of the mathematical models, minimizing the risk of lack of fit. The response variables encompassed cooking time, weight gain, solid loss, firmness, fracturability, and instrumental color components (refer to Section 2.9). The desirability function was employed to optimize response variables within specified ranges, assigning a level of importance and an objective (maximize, minimize, or maintain within range) to each response variable. The experimental data were analyzed using the statistical package Design-Expert V.11.0.1 (Stat-Ease Inc., Minneapolis, MN, USA). Design-Expert facilitated the solution of the second-order polynomial regression equation.

### 2.4. Cooking Time

Cooking time was assessed by cooking 10 g of the sample in 140 mL of boiling water without salt until reaching an appropriate visual quality. The cooked sample underwent compression every 30 s between two slides until the central axis disappeared, following the AACC method 16-50 [21].

### 2.5. Weight Gain

The weight gain of the pasta was determined following the procedure outlined by Cleary and Brennan [22], with the following adaptations: 100 g of pasta was cooked in 1 L of water without salt for the optimal cooking time (5 min) and subsequently cooled in 2 L of cold water. The pasta was then dried with absorbent paper and weighed using an analytical balance. To calculate the percentage of the total weight gain of cooked pasta, the following equation was applied:

Weight gain = (cooked pasta weight − raw pasta weight/raw pasta weight) × 100

### 2.6. Solids Loss

The percentage of solids lost in the cooking water was calculated by evaporating 25 mL of the cooking water, obtained during the mass increase analysis, in an oven at 105 °C until a constant mass was reached, in accordance with AACC method 16–50 [21].

### 2.7. Firmness

The pasta was cooked based on the optimal cooking time, and the firmness analysis (N) was conducted following method 66–50.01 [23], utilizing 6 pasta strands for each of the 10 replicates in each test. The testing conditions comprised probe A/LKB-F, pre-test and test speeds set at 0.50 mm/s, and a post-test speed at 10.0 mm/s, with a height of 15 mm, a distance of 14 mm, and a force threshold of 0.049 N.

### 2.8. Fracturability

Fracturability assessment utilized the 3-Point Bending Rig (HDP/3PB) with test speeds set at 0.50 mm/s, a height of 20 mm, a 5 mm compression mode distance, and a force threshold of 0.049 N. The analysis involved 10 replicates, with each replicate consisting of 1 pasta strand measuring 50 mm in length, following the methodology outlined by Ungureanu-Iuga, Dimian, and Mironeasa [24].

### 2.9. Instrumental Color

The CIELAB color was measured in triplicate, employing a colorimeter (Minolta, CR-310, Osaka, Japan). Within the CIE color space, L* denotes lightness, a* represents greenness/redness, and b* indicates blueness/yellowness of the dry pasta.

### 2.10. Statistics

The preparation of pasta and the physicochemical analysis of samples were carried out in duplicate. The results were expressed as mean ± standard deviation. Differences among the studied parameters were assessed via a one-way analysis of variance (ANOVA). Post hoc testing using the Bonferroni method was employed to distinguish between mean values with 95% confidence intervals. Regression models for cooking time, weight gain, loss of solids, firmness, fracturability, and CIELAB color (L*, a*, b*) were established using Design Expert 10 (Stat-Ease Inc., Minneapolis, MN, USA). ANOVA regression models were employed to select the most significant model (*p* < 0.05) and the best fit (R^2^ > 0.80). Response surfaces and a desirability methodology were utilized to identify optimal formulations.

## 3. Results and Discussion

### 3.1. Effect of Replacing WF with Sprouted Pseudocereal Flour and Cushuro Powder on Cooking Time of Pasta

The effect of replacing WF with sprouted pseudocereal flour and CuP on cooking time, solids loss, weight gain, firmness, fracturability, and instrumental color is shown in Table 3. The results indicated that as the substitution of WF increases, the cooking time also increases and reaches a peak when the substitution of SQF and CuP is 13% in both cases in PQC, or when the substitution of SKF is 8% and CuP is 13%, specifically in PKC. According to Boukid et al. [25], the softening during cooking is due to the penetration of water and the gelatinization of starch, which starts from the surface and progresses towards the center of the pasta.

In the present study, the cooking time for the control sample (100% WF) was 6 min, i.e., lower than that obtained in the other pasta formulations. This differs from the findings reported by Xing et al. [26], where pastas with SQF substitution had a shorter cooking time compared to 100% wheat pasta. It is worth noting that the control sample had a cooking time of approximately 5 min, a value similar to that obtained in this research. The increase in cooking time could be attributed to the incorporation of CuP. Similarly, Vedia et al. [27] reported cooking times higher than their control sample (13.33 ± 1.51 min) in pastas with partial substitution of raw kiwicha flour, ranging from 15.67 ± 0.57 to 20.00 ± 1.00 min. Granito and Ascanio [28] suggest that increasing the fiber content leads to an increased cooking time due to competition between the fiber and the starch for water. Similar to the pasta with SQF, these formulations increased the fiber content through SKF (23.06 ± 0.67% total dietary fiber) and CuP (19.77 ± 0.57% total dietary fiber) [19].

### 3.2. Effect of Replacing WF with Sprouted Pseudocereal and Cushuro Flours on Solids Loss and Weight Gain of Pasta

The increase in pasta weight is related to the water absorption capacity, which is a quality parameter of great importance since it is not only of interest to consumers, but also reflects the content and quality of the protein and can reflect the pasta yield [27]. Regarding weight gain, as the substitution with sprouted quinoa and cushuro powder is greater, the weight gain also increases. Similar results showed that water absorption was greater in pastas with legume flour replacement, varying from 171% in wheat semolina pasta to 185% in pastas with substitution of wheat semolina by 10% of cowpea (*Vigna sinensis* L.) flour [29].

The loss of solids is an indicator of pasta quality, considering it as the resistance to disintegration by cooking in water [30]. In a good quality pasta, the loss of solids due to cooking should be less than 10% [26]. Research indicates that when there is a greater amount of starch and a protein network that is not very well structured, the absorption of water is difficult and the dispersion of solids (mainly amylose) is facilitated [31]. Likewise, when the protein content increases, a more closed network is formed in the dough that does not allow for solids to escape into the cooking water. In general, by including fiber-rich products in the pasta, this produces an increase in the loss of solids and an increase in water absorption [32].

In general, the partial use of non-gluten protein flours results in the weakening of the protein network that surrounds the starch granules, reducing cooking time and promoting weight gain, favoring solid loss. This behavior was not observed in our study. However, Bresciani et al. [33] report that pasta processing can drastically affect the quality characteristics of the pasta. The use of appropriate unit operations, such as milling, mixing, kneading, extrusion, and drying, can mitigate the deleterious effects that the use of unconventional flours may have on the characteristics of the final product.

### 3.3. Effect of Replacing WF with Sprouted Pseudocereal Flours and Cushuro Powder on Firmness and Fracturability of Pasta

The firmness increases as the concentration of cushuro flour decreases and decreases as the concentration of sprouted quinoa flour increases. With the use of germinated flours (especially in higher concentrations), there is a higher presence of amylolytic and proteo-lytic enzymes, resulting in the weakening of the gluten network [26]. During pasta production, the drying stage plays an important role, since, due to heat, the protein coagulates, forming a protein network around the starch granules with enhanced strength and firmness, thus preventing the starch granules from passing into the cooking water [4]. Similarly, pasta made with substitution of quinoa and spinach flour obtained less firmness than the control sample (only wheat flour) in such a way that the samples with 10% substitutions indicated better firmness than samples with 20–30% substitution [6].

As with pastas with sprouted quinoa flour, as the substitution of sprouted Kiwicha flour increases, the pasta loses firmness. Likewise, it was reported that pasta with a higher percentage of wheat flour had greater firmness because the gluten it contains favors the formation of a firmer structure in such a way that pasta with less cooking residue and a better yellow color is obtained. However, this last characteristic is not notable due to the dark green color provided by the cushuro in this research. Kiwicha flour has a higher content of dietary fiber compared to quinoa, which directly influenced the cooking characteristics and texture of the pasta [34]. In addition to the fiber content, the particle size of the non-starchy polysaccharide can directly influence texture properties. Generally, it is observed that smaller particle sizes result in pastas with greater firmness and less stickiness [33].

### 3.4. Effect of Replacing WF with Sprouted Pseudocereal Flours and Cushuro Powder on Instrumental Color of Pasta

The response surface and contour plots depicting the behavior of the three color components reveal distinct trends based on the composite flour formulation. Notably, the L component (brightness) is consistently lower across all PQC and PKC formulations compared to the control pasta. Moreover, an increase in the percentage of cushuro flour corresponds to a reduction in both yellowness (b) and redness (a), with the latter intensifying as the cushuro content decreases. Both in PQC and PKC, an increase in the percentage of cushuro flour results in reduced yellowness (b) and redness (a), attributed to the inherently dark color of this cyanobacterium. Corpus et al. [14] classify cushuro into three colors: brown, light green, and dark green, with corresponding component values corresponding to dark green (L = 8.35 ± 0.26; a = −3.75 ± 0.06; b = 3.98 ± 0.38). This classification explains the darkening observed in the pasta.

**Figure 2:**
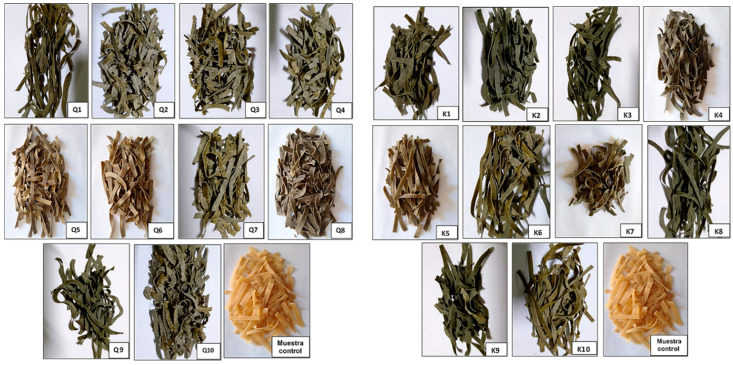
On the (**left**), pasta with partial replacement of wheat flour with sprouted quinoa flour and cushuro powder. On the (**right**), pasta with partial replacement of wheat flour with sprouted kiwicha flour and cushuro powder.

These findings align with the outcomes reported by Xing et al. [26], demonstrating a shift in pasta color from a whitish hue to a pale brown with an escalating substitution of SQF. Additionally, it was observed that both a and b values increased within the 10 to 30% SQF substitution range for pasta (*p* > 0.05). In a related context, Xing et al. [26] conducted experiments involving partial substitution of native and germinated quinoa flour in pasta, revealing a noticeable decline in luminosity compared to the control sample composed solely of wheat semolina. Lux et al. [35] investigated the impact of adding kiwicha flour to pasta, concluding that higher percentages of WF and reduced concentration of kiwicha flour result in pasta with improved color (enhanced yellowness index and lightness), as well as enhanced firmness. Similar outcomes were noted by Cárdenas-Mendoza et al. [32], where the addition of kiwicha flour to pasta led to decreased luminosity and a shift of the “a” component towards reddish tones. This shift is primarily attributed to the Maillard reaction induced by temperature, causing a subtle darkening of the pasta (Figure 2).

### 3.5. Optimal Supplementation Ratio of WF with Sprouted Pseudocereal Flour and Cushuro Powder to Improve Texture and Color of Pasta

Modeling the impact of substituting refined wheat flour (WF) with sprouted pseudocereals and cushuro on cooking time, solids loss, weight gain, firmness, fracturability, and instrumental color in pasta involves developing a mathematical representation of the relationship between the substitution ratio and these variables. This can be achieved using statistical modeling techniques, such as regression analysis or mathematical modeling.

**Figure 3:**
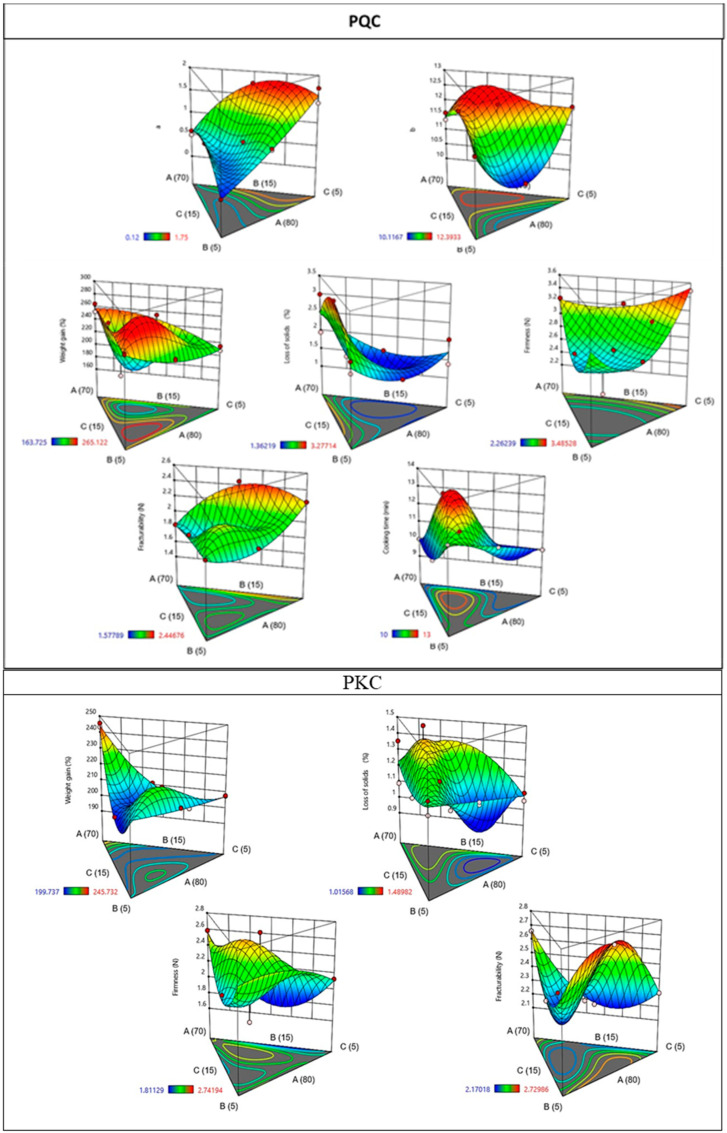
Effect of composite flour formulation on PQC and PKC.

The study delved into the variations in cooking time, weight gain, solids loss, firmness, fracturability, and instrumental color based on the composite flour formulation. To comprehend the impact of independent variables, the results were visually represented in Figure 3. For each dependent variable, the influential factors with a significant effect (*p* < 0.05) were fine tuned to create the most effective mathematical model, capable of predicting the behavior of the dependent variable (see Table 4). This comprehensive statistical modeling approach establishes a robust foundation for both understanding and optimizing the composite flour formulation concerning pasta cooking quality, texture, and color.

Table 4 shows the predictive regression models for the technological parameters of cooking time, weight gain, loss of solids, instrumental color, firmness, and breaking strength, obtaining statistically significant results in pasta with germinated quinoa for luminosity, weight gain, loss of solids, and breaking strength. In the pasta with partial replacement of kiwicha flour, results of *p* > 0.05 were obtained for the parameters of chromaticity, cooking time, loss of solids, and firmness.

To find the optimal formulation of composite flour for the enhancement of weight gain, firmness, color, reducing cooking time, solid loss, and fracturability, a multiple response optimization was performed. This procedure helped in determining the combination of WF, SQF/SKF, and CuP ratios, giving equal importance to all responses (Table 5). The optimum value reached for desirability was 0.63 for PQC. The optimal formulation provided in the multiple response optimization for PQC was 79% WF, 13% SQF, and 8% CuP, for which the predicted values for L*, a*, b*, cooking time, weight gain, solid loss, firmness, and fracturability were 62.56, −0.13, 12.16, 8.03 min, 244.07%, 1.23%, 2.57 N, and 2.66 N, respectively. Regarding PKC, the optimal formulation was 70% WF, 15% SKF, and 15% CuP (optimum desirability value = 0.68), for which the predicted values reached 62.95, 1.27, and 11.27 for the L*, a* and b* color components, 10 min for cooking time, 235.59% for weight gain, 1.44% for solid loss, 2.78 N for firmness, and 2 N for fracturability [14].

## 4. Conclusions

The partial substitution of wheat flour with sprouted pseudocereal grains and cushuro in the pasta formulation presented acceptable characteristics compared to the technological parameters of the control sample, which represents the use of these alternative flours as a promising opportunity in the production of pasta. The optimization of flour mixtures using a simple centroid mixture design and an additional multiple response desirability method allowed for obtaining pastas with a technological quality close to the desirable values of the control pasta regarding texture properties (fracturability and firmness). For both PQC and PKC pastas, there was a substantial increase in cooking-related weight gain and a slight rise in leached solids content during the cooking step, but these results seem to reflect appropriate values when it comes to integral pasta. The color of pasta with quinoa and kiwicha was altered, with less yellowish and darker shades, linked of the characteristic of whole grains, which are associated with healthy products by consumers. All these results encourage manufacturers to make wider use of SQF and SKF to create and promote healthier versions of cereal-based products, accelerating the transition towards healthy and sustainable food systems. Further research can enhance these findings. Evaluating nutritional stability and conducting sensory evaluations during and after storage will provide insights into the pasta’s overall quality. Microbiological analyses are crucial to ensure the safety of the pasta, especially when introducing novel ingredients. Additionally, considering sustainable packaging options aligns with the global trend towards eco-friendly practices, contributing to the promotion of healthier and more sustainable food systems. These comprehensive studies will not only support the successful integration of alternative flours in pasta, but also inform manufacturers and consumers about the product’s long-term storage.

## Figures and Tables

**Table 1:** Simplex Centroid Mixture Design for PQC.

Test	Flour Blend Ratio		
	WF	SQF	CuP
1	70	15	15
2	80	5	15
3	80	15	5
4	75	10	15
5	75	15	10
6	80	10	10
7	76	12	12
8	74	13	13
9	79	8	13
10	79	13	8
11	80	15	5
12	80	10	10
13	70	15	15
14	80	5	15

Abbreviations: WF, wheat flour; SQF, sprouted quinoa flour; CuP, cushuro powder.

**Table 2:** Simplex Centroid Mixture for PKC.

Test	Flour Blend Ratio		
	WF	SKF	CuP
1	70	15	15
2	80	5	15
3	80	15	5
4	75	10	15
5	75	15	10
6	80	10	10
7	76	12	12
8	74	13	13
9	79	8	13
10	79	13	8
11	80	15	5
12	80	10	10
13	70	15	15
14	80	5	15

Abbreviations: WF, wheat flour; SKF, sprouted kiwicha flour; CuP, cushuro powder.

**Table 3:** Effect of flour formulation on cooking time, weight gain, solids loss, firmness, fracturability, and instrumental color.

Pasta Type	Recipe No.	Ingredient Ratio			Cooking Time (min)	Weight Gain (%)	Loss of Solids (%)	Firmness (N)	Fracturability (N)	Instrumental Color		
		SQF/SKF	CuP	WF						L*	a*	b*
PQC	1	15	15	70	10	265.12	1.96	3.25 ± 0.33	1.83 ± 0.22	62.95 ± 0.03	0.59 ± 0.03	11.34 ± 0.05
	2	5	15	80	12	250.34	2.33	3.09 ± 0.27	1.95 ± 0.30	67.22 ± 0.07	0.12 ± 0.07	11.55 ± 0.07
	3	15	5	80	10	214.02	1.38	3.49 ± 0.18	2.25 ± 0.28	61.72 ± 0.07	1.73 ± 0.07	12.04 ± 0.10
	4	10	15	75	10	262.97	3.28	2.70 ± 0.28	1.96 ± 0.34	66.66 ± 0.07	0.76 ± 0.07	12.23 ± 0.05
	5	15	10	75	10	254.78	1.60	3.23 ± 0.22	2.45 ± 0.25	62.71 ± 0.04	1.75 ± 0.04	12.05 ± 0.05
	6	10	10	80	11	218.63	1.43	2.68 ± 0.26	1.87 ± 0.20	68.61 ± 0.04	0.77 ± 0.04	10.15 ± 0.04
	7	12	12	76	12	254.46	1.41	2.71 ± 0.20	2.15 ± 0.28	65.41 ± 0.05	0.72 ± 0.05	12.34 ± 0.05
	8	13	13	74	13	163.73	1.53	2.55 ± 0.20	1.58 ± 0.17	63.85 ± 0.03	0.55 ± 0.03	12.39 ± 0.05
	9	8	13	79	12	259.80	2.11	2.26 ± 0.12	2.00 ± 0.20	62.88 ± 0.08	0.28 ± 0.08	10.73 ± 0.03
	10	13	8	79	10	200.50	1.36	3.08 ± 0.17	1.87 ± 0.23	60.23 ± 0.02	1.48 ± 0.02	11.58 ± 0.04
	11	15	5	80	10	207.32	2.05	3.49 ± 0.18	2.25 ± 0.28	66.75 ± 0.04	1.42 ± 0.04	12.09 ± 0.04
	12	10	10	80	11	216.44	1.43	2.68 ± 0.26	1.87 ± 0.20	68.62 ± 0.11	0.74 ± 0.11	10.12 ± 0.04
	13	15	15	70	10	252.65	3.00	3.25 ± 0.33	1.83 ± 0.22	63.54 ± 0.03	0.50 ± 0.03	11.58 ± 0.04
	14	5	15	80	12	248.02	2.04	3.09 ± 0.27	1.95 ± 0.30	67.23 ± 0.02	0.12 ± 0.02	11.52 ± 0.03
PKC	1	15	15	70	8	243.39	1.39	2.59 ± 0.41	2.20 ± 0.31	62.04 ± 0.01	−0.097 ± 0.13	12.08 ± 0.03
	2	5	15	80	8	214.35	1.26	2.44 ± 0.16	2.51 ± 0.21	65.59 ± 0.02	−0.58 ± 0.01	10.79 ± 0.02
	3	15	5	80	8	207.20	1.05	2.12 ± 0.13	2.30 ± 0.20	63.17 ± 0.01	−0.79 ± 0.01	11.52 ± 0.01
	4	10	15	75	9	201.01	1.13	2.05 ± 0.19	2.32 ± 0.21	64.38 ± 0.01	0.14 ± 0.01	12.45 ± 0.02
	5	15	10	75	8	209.24	1.35	1.81 ± 0.12	2.17 ± 0.28	64.82± 0.57	0.02 ± 0.02	13.33 ± 0.05
	6	10	10	80	10	210.56	1.13	2.44 ± 0.16	2.73 ± 0.28	65.92 ± 0.03	0.33 ± 0.02	12.74 ± 0.04
	7	12	12	76	10	219.39	1.04	2.74 ± 0.30	2.29 ± 0.14	66.10 ± 0.02	0.15 ± 0.01	13.40 ± 0.02
	8	13	13	74	10	199.74	1.49	2.34 ± 0.24	2.28 ± 0.26	62.48 ± 0.02	−0.31 ± 0.01	11.64 ± 0.04
	9	8	13	79	11	213.52	1.29	1.86 ± 0.16	2.41 ± 0.21	61.51 ± 0.02	0.13 ± 0.02	12.26 ± 0.05
	10	13	8	79	10	201.61	1.02	2.05 ± 0.20	2.66 ± 0.22	67.90 ± 0.02	0.21 ± 0.01	14.31 ± 0.01
	11	15	5	80	8	208.41	1.10	2.12 ± 0.13	2.30 ± 0.20	67.20 ± 0.02	0.17 ± 0.01	13.44 ± 0.04
	12	10	10	80	10	211.29	1.15	2.44 ± 0.16	2.73 ± 0.28	67.32 ± 0.01	0.22 ± 0.02	12.75 ± 0.04
	13	15	15	70	8	245.73	1.35	2.59 ± 0.41	2.20 ± 0.31	62.84 ± 0.15	−0.07 ± 0.01	12.65 ± 0.02
	14	5	15	80	8	215.01	1.18	2.44 ± 0.16	2.51 ± 0.21	63.39 ± 0.02	−0.54 ± 0.02	10.47 ± 0.02
Control	15	0	0	100	6	114.75	0.47	2.68 ± 0.24	2.56 ± 0.30	81 ± 0.05	0.89 ± 0.05	14.79 ± 0.03

Abbreviations: CuP: cushuro flour; PKC, wheat-based pasta supplemented with sprouted kiwicha and cushuro flour; PQC, wheat-based pasta supplemented with sprouted quinoa and cushuro powder; SKF, sprouted kiwicha flour; SQF, sprouted quinoa flour; WF: refined wheat flour.

**Table 4:** Predictive regression models for cooking time, solids loss, weight gain, firmness, fracturability, and instrumental color of pasta according to the independent variables [WF (A), SQF/SKF (B), and CuP (C) ratios in the composite flour].

Pasta Type	Dependent Variables	Mathematical Models	*p*-Value	R^2^ (pred)	R^2^ (adj)
PQC	L	62.92A + 67.64B + 63.92C	0.387	0.68	0.18
	a	0.54A + 0.12B + 1.57C + 1.67AB + 2.72AC − 0.32BC − 42.57A^2^BC − 20.75AB^2^C + 20.47ABC^2^	0.0004	0.98	0.96
	b	11.44A + 11.52B + 12.04C + 2.71AB + 0.94AC − 6.73BC + 69.31A^2^BC − 45.6AB^2^C + 34.08ABC^2^	0.0002	1	0.99
	Cooking time	10.02A + 12.02B + 10.02C − 3.76AB + 0.24AC + 0.08BC + 246.52A^2^BC + 31.96AB^2^C − 112.04ABC^2^	0.001	0.98	0.94
	Weight gain	257.05A + 247.35B + 208.84C + 13.75AB + 58AC − 56.92BC − 6866.1A^2^BC + 5068.38AB^2^C + 344.41ABC^2^	0.228	0.76	0.39
	Solid loss	2.48A + 2.18B + 1.71C + 3.73AB − 2.05AC − 2.08BC − 71.74A^2^BC + 7.52AB^2^C + 4.05ABC^2^	0.116	0.83	0.56
	Firmness	3.27A + 3.06B + 3.53C − 2.55AB − 0.78AC − 2.60BC	0.002	0.87	0.78
	Fracturability	1.82A + 1.94B + 2.23C + 0.13AB + 1.48AC − 0.97BC − 31.02A^2^BC + 30.50AB^2^C − 11.86ABC^2^	0.185	0.79	0.45
PKC	L	62.56A+ 64.58B + 66.50C	0.089	0.35	0.24
	a	−0.13A − 0.53B − 0.28C + 1.38AB + 0.41AC + 2.77BC	0.154	0.58	0.32
	b	12.16A + 10.63B + 12.65C + 2.95AB + 3.76AC + 5.26BC	0.074	0.66	0.45
	Cooking time	8.02A + 9.08B + 8.03C + 2.65AB + 0.37AC + 2.23BC + 51.04ABC	0.109	0.63	0.32
	Weight gain	244.07A + 214.20B + 206.85C − 120.23AB − 72.58AC − 4.24BC − 924.26A^2^BC + 1562.54AB^2^C + 244.85ABC^2^	0.018	0.93	0.81
	Solid loss	1.38A + 1.22B + 1.08C − 0.57AB + 0.57AC − 0.006BC + 14.15A^2^BC + 5.67AB^2^C − 26.13ABC^2^	0.336	0.71	0.24
	Firmness	2.57A + 2.42B + 2.10C − 2.06AB − 2.38AC + 0.57BC + 46.56A^2^BC − 23.99AB^2^C + 17.53ABC^2^	0.207	0.78	0.41
	Fracturability	2.66A + 2.51B+ 2.30C − 1.01AB − 1.19AC + 1.33BC −13.97A^2^BC − 14.22AB^2^C + 25.62ABC^2^	0.001	0.98	0.94

Abbreviations: PKC, wheat-based pasta supplemented with sprouted kiwicha and cushuro flour; PQC, wheat-based pasta supplemented with sprouted quinoa and cushuro powder.

**Table 5:** Composition of flour blends and predicted values for cooking time, solids loss, weight gain, firmness, fracturability and instrumental color in pasta at optimum desirability value (D).

Pasta Type	Optimum Desirability Value (D)	Flour Formulation at Optimum D	Response Variables	Predicted Values	−95% CI	+95% IQ
PQC	0.63	WF: 79% SQF: 13% CuP: 8%	L*	62.95	58.40	67.51
			a*	1.27	0.99	1.54
			b*	11.27	11.10	11.43
			Cooking time (min)	10	9.39	10.61
			Weight gain (%)	235.59	178.14	293.05
			Solid loss (%)	1.44	0.46	2.42
			Firmness (N)	2.78	2.60	2.95
			Fracturability (N)	2	1.60	2.39
PKC	0.68	WF: 70% SKF: 15% CuP: 15%	L*	62.56	60.20	64.92
			a*	−0.13	−0.59	0.33
			b*	12.16	10.90	13.42
			Cooking time (min)	8.03	6.52	9.53
			Weight gain (%)	244.07	232.91	255.24
			Solid loss (%)	1.23	1.01	1.45
			Firmness (N)	2.57	2.17	2.97
			Fracturability (N)	2.66	2.58	2.75

Abbreviations: CI: confidence interval; CuP: cushuro powder; PKC: wheat-based pasta supplemented with sprouted kiwicha and cushuro powder; PQC: wheat-based pasta supplemented with sprouted quinoa and cushuro powder; SKF: sprouted kiwicha flour; SQF: sprouted quinoa flour; WF: refined wheat flour.

## Data Availability

Data is contained within the article.
